# Lactate Ringer’s anti-inflammatory effects in acute pancreatitis are potentially mediated by protein lactylation of extracellular vesicles

**DOI:** 10.1038/s41598-026-56277-z

**Published:** 2026-06-06

**Authors:** Olga Armengol-Badia, Jaxaira Maggi, Lucía Guilabert, Enrique de-Madaria, Montserrat Carrascal, Daniel Closa

**Affiliations:** 1 Department Experimental Pathology, IIBB-CSIC-IDIBAPS, c/ Rosselló, 161, 7, 08036 Barcelona, Spain; 2 Facultat de Farmàcia i Ciències de l’Alimentació, Universitat de Barcelona, Barcelona, Spain; 3 Biological and Environmental Proteomics, IIBB-CSIC-IDIBAPS, Barcelona, Spain; 4 Gastroenterology Department, Dr. Balmis General University Hospital-Instituto de Investigación Sanitaria y Biomédica de Alicante (ISABIAL), Alicante, Spain; 5 Centro de Investigación Biomédica en Red de Enfermedades Hepáticas y Digestivas (CIBEREHD), Instituto de Salud Carlos III, Madrid, Spain; 6 Department of Clinical Medicine, Miguel Hernández University, Elche, Spain; 7 CSIC/UAB Proteomics Laboratory, Autonomous University of Barcelona, Cerdanyola del Vallès, Spain

**Keywords:** Pancreatitis, Lactated Ringer’s, Lactylation, Extracellular vesicles, Diseases, Immunology, Medical research

## Abstract

Lactated Ringer’s solution (LR) has been associated with anti-inflammatory effects in acute pancreatitis, likely attributable to its lactate content, but the underlying mechanisms remain poorly understood. Circulating small extracellular vesicles (sEVs) contribute to systemic inflammation in this condition by activating macrophages, and we investigated whether lactate could modulate their inflammatory activity. sEVs derived from BxPC-3 pancreatic cells were treated with lactate and their inflammatory potential assessed by measuring their capacity to induce pro-inflammatory cytokine expression in macrophages. Circulating sEVs were also isolated from patients with acute pancreatitis receiving either LR or normal saline and analysed using the same approach. Vesicle uptake and protein lactylation levels were evaluated by microscopy and western blot, respectively. Lactate treatment reduced sEV-induced macrophage activation without altering vesicle uptake, and was associated with a modest increase in protein lactylation. A similar reduction in inflammatory activity was observed in circulating sEVs from LR-treated patients, but not in those receiving normal saline, alongside a comparable increase in protein lactylation. These findings suggest that lactate can directly modify sEV activity and that the anti-inflammatory effects of LR in acute pancreatitis may be mediated, at least in part, through lactylation-dependent modulation of sEV inflammatory activity.

**Supplementary Information:**

The online version contains supplementary material available at 10.1038/s41598-026-56277-z.

## Introduction

Lactate is a physiological metabolite with regulatory functions in immune and inflammatory processes^1^. These properties are also relevant in clinical contexts in which systemic lactate levels are deliberately modified, such as during the administration of lactated Ringer’s solution (LR), a crystalloid fluid widely used in resuscitation and critical care^2^. Infusion of LR increases circulating lactate concentrations and has been reported to exert anti-inflammatory effects compared with normal saline (NS). In acute pancreatitis, treatment with LR has been associated with a reduced inflammatory response, an effect not observed following NS administration^3^. However, despite these clinical observations, the molecular mechanisms underlying these effects remain poorly understood.

Protein lactylation is a recently identified post-translational modification that represents a novel mechanism by which lactate can influence protein structure and function^4^. By covalently modifying lysine residues, lactylation has been associated with alterations in gene regulation and immune responses, expanding the recognized biological roles of lactate beyond its classical metabolic functions^5^. Initially identified on histones, this modification has since been detected on multiple non-histone proteins, suggesting that lactylation may affect a wide range of intracellular and extracellular processes. The relevance of protein lactylation becomes particularly evident in physiological and pathological conditions characterized by elevated lactate concentrations.

On the other hand, recent evidence highlights small extracellular vesicles (sEVs) as key mediators of systemic inflammation in acute pancreatitis, contributing to immune activation and organ dysfunction^6,7^. Given the ability of lactate to induce protein lactylation, we hypothesized that the anti-inflammatory properties attributed to LR may be related to lactylation events within circulating sEVs, thereby modulating their inflammatory potential. Such modifications could affect both luminal and surface-associated proteins^8^, potentially altering vesicle–cell interactions and biological activity.

Here, we investigated how lactate exposure modifies the inflammatory activity of sEVs and whether changes in their protein lactylation may contribute to these effects, in the context of LR administration during acute pancreatitis.

## Methods

### Cells

Human THP-1 cells were cultured in suspension in RPMI 1640 medium supplemented with 10% fetal calf serum (FCS), 2 mM L-glutamine, 100 U/mL penicillin, and 100 µg/mL streptomycin. Cells were differentiated into macrophages by incubation with 100 nM phorbol 12-myristate 13-acetate (PMA; Sigma-Aldrich) for 48 h. Subsequently, the PMA-containing medium was removed and replaced with fresh PMA-free medium, and cells were allowed to recover for an additional 24 h. Cultures were maintained at 37 °C in a humidified atmosphere containing 5% CO₂.

Human pancreatic BxPC-3 cells were cultured in Dulbecco’s modified Eagle’s medium (DMEM) supplemented with 10% FCS, 2 mM L-glutamine, 100 U/mL penicillin, and 100 µg/mL streptomycin. Cells were maintained at 37 °C in a humidified atmosphere containing 5% CO₂. For small extracellular vesicle (sEV) isolation, cells were seeded in 175 cm² flasks and grown to approximately 80% confluence. The culture medium was then replaced with serum-free DMEM supplemented with 2 mM L-glutamine, 100 U/mL penicillin, and 100 µg/mL streptomycin, and cells were incubated for 48 h. Conditioned media were subsequently collected and processed for sEV purification.

In selected experiments, sEV production was performed under the same conditions with the addition of sodium lactate (28mM) to the culture medium.

### Patients

This study was conducted in accordance with Good Clinical Practice guidelines and the ethical principles for medical research involving human subjects outlined in the Declaration of Helsinki^8^. The study was reviewed and approved by the Ethics Committee from Dr Balmis General University Hospital (Comité Ético de Investigación con Medicamentos, CEIM) with reference number 2023-031, approved on 09/03/2023 (Acta nº 2023/02). The collection of samples from patients with acute pancreatitis was performed prospectively, and written informed consent was obtained from all participants.

Plasma samples were collected from patients diagnosed with acute pancreatitis who received either LR (*n* = 10) or NS (*n* = 10) as fluid therapy and were stored at − 70 °C until analysis. The diagnosis of acute pancreatitis was established based on the presence of at least two of the following criteria: serum amylase and/or lipase levels exceeding three times the upper limit of normal, imaging findings consistent with acute pancreatitis, and/or typical abdominal pain. The final severity of AP was categorized retrospectively according to the revised Atlanta classification system as mild, moderately severe or severe^9^. Exclusion criteria included infections, pregnancy and time between the onset of symptoms and blood collection greater than 48 h. Clinical characteristics of patients are included in Table 1.

**Table 1:** Clinical characteristics of patients from the two groups included in the study.

Fluid type	Normal saline (SS) (*n* = 10)	Ringer lactate (RL) (*n* = 10)
Age (years)	58 (43–102)	68 (48–96)
Gender (male)	7	2
Risk factors and comorbidities		
Smoker	3	1
Alcohol abuse	3	0
Hypertension	4	4
Diabetes mellitus	2	1
Chronic kidney disease	0	3
Etiology		
Biliary	6	7
Alcohol	2	0
TG	0	1
Drug	0	1
Idiopathic	2	1
Severity		
Mild	7	8
Moderate	1	1
Severe	2	1
Outcomes		
ICU admission	1	0
In-hospital mortality	2	0

### Small extracellular vesicle (sEV) isolation

Small extracellular vesicles (sEVs) were isolated from cell culture conditioned media and human plasma using differential centrifugation and ultracentrifugation, with minor modifications from previously described protocols^6^.

*sEV isolation from cell culture conditioned media*: Conditioned media were sequentially centrifuged at 2,000 × g for 10 min and 10,000 × g for 30 min to remove cells and cellular debris. The resulting supernatant was filtered through a 0.22 μm pore-size filter and subjected to ultracentrifugation at 120,000 × g for 180 min. The pellet was resuspended in phosphate-buffered saline (PBS) and ultracentrifuged again at 120,000 × g for 180 min for washing. The final sEV pellet was resuspended in PBS for downstream analyses.

*sEV isolation from human plasma*: Plasma samples (1 mL) were centrifuged at 2,000 × g for 10 min at 4 °C to remove cells and debris. Following this initial step, samples were treated with thrombin (5 U/mL; Sigma, St. Louis, MO, USA) for 5 min to remove fibrin, and centrifuged at 10,000 × g for 30 min. The fibrin pellet was discarded, and the resultant supernatant was diluted in PBS to a final volume of 25 mL, filtered through a 0.22 μm pore-size filter, and ultracentrifuged at 120,000 × g for 180 min. The pellet was resuspended in PBS and subjected to a second ultracentrifugation step at 120,000 × g for 180 min. The final sEV pellet was resuspended in PBS for downstream analyses.

Isolated sEVs were quantified by measuring total protein content using the Pierce BCA Protein Assay Kit (Thermo Fisher Scientific, Waltham, MA, USA).

Small extracellular vesicles (sEVs) previously isolated from BxPC-3 pancreatic cells were incubated with 28 mM sodium lactate (Sigma). This concentration was selected to match the lactate concentration present in LR. Vesicles were incubated for 1 h at room temperature (RT). Excess, unbound lactate was removed by repeated centrifugal filtration and washing steps using 300 kDa molecular weight cut-off Nanosep filter devices (Pall Corporation), which retain sEVs while allowing free lactate to pass through the membrane. This procedure yielded lactate-treated sEVs (Lac-sEVs).

The same procedure was used to treat sEVs with individual lactate stereoisomers. Because’s LR contains a mixture of the two lactate stereoisomers, sEVs were incubated with 14 mM of either L-lactate or D-lactate (Sigma) to match the effective contribution of each isomer. Incubation with L-lactate generated L-Lac sEVs, whereas incubation with D-lactate generated D-Lac sEVs.

### Nanoparticle tracking analysis

Quantification of concentration and size of sEV obtained in plasma and cell culture medium were carried out using a NanoSight LM10 machine (NanoSight) by the ICTS “NANBIOSIS” at the ICMAB-CSIC, as previously described^7^. All the parameters of the analysis were set at the same values for all samples and three 1 min-long videos were recorded in all cases. The background was measured by testing filtered PBS, which revealed no signal.

### SDS-PAGE and western blot

sEV proteins were extracted in RIPA buffer supplemented with protease inhibitors. Proteins were separated on a 4–12% SDS-PAGE (Invitrogen™, NuPAGE™ 4 to 12%, Bis-Tris, 1.0–1.5 mm, Thermo Fisher, Waltham, MA, USA), transferred onto a PVDF membrane (Immuno-Blot, Bio Rad, Hercules, CA, USA) and blocked for 1 h in 5% nonfat milk in TBST, followed by overnight incubation at 4 °C with antibodies against TSG101 (14497-1-AP), ALIX (12422-1-AP), CD9 (20597-1-AP), Calnexin (10427-2-AP) (ProteinTech, Rosemont, IL, USA) and L-Lactyllysine (PTM-1425)(PTM-Bio, Chicago, IL). The blots were washed and incubated for 1 h at room temperature with DyLight 800-conjugated secondary antibodies (925-32211) (LI-COR Biosciences, Lincoln, NE, USA). Immunoreactive bands were visualized using an Odyssey Infrared Imaging System, Image Lab Touch Software version 2.3.0.07 (LI-COR Biosciences, Lincoln, NE, USA)^6^.

### RNA isolation and qPCR

Total RNA from cells was extracted using the TRizol^®^ reagent (Invitrogen, Carlsbad, CA). RNA was quantified by measuring the absorbance at 260 and 280 nm using a NanoDrop ND-1000 spectrophotometer (NanoDrop Technologies, USA). cDNA was synthesized from 1 µg RNA sample using the iScript cDNA synthesis kit (Bio-Rad, CA, USA).

Subsequent quantitative polymerase chain reaction (qPCR) was performed in a DNA Engine, Peltier Thermal Cycler (Bio-Rad, CA, USA) using iTaq™ Universal SYBR^®^ Green Super mix and the corresponding primers. Reactions were performed in duplicate and threshold cycle values were normalized to *GAPDH* gene expression. The specificity of the products was determined by melting curve analysis. The relative expression of target genes to *GAPDH* was calculated by the ΔC(t) formula^7^.

### sEV staining and cell uptake

For uptake experiments, sEV were labeled with PKH26 Red Fluorescent Cell Linker Dye (Sigma, St Louis, MO) according to the supplier’s specifications. The staining reaction was stopped with 3% BSA for 1 min, and the labeled vesicles were washed three times with PBS in order to remove the unbound dye, using 300 KDa Nanosep centrifugal devices (Pall Corporation). For cell uptake analysis, 5ug/mL of PKH26 red-stained sEVs and Lac sEVs were added to the diferent cell cultures for 3 h^7^. Macrophage differentiated THP1 cells were stained with green PKH67 (Sigma, St Louis, MO) using the same protocol. The cell nucleus was stained blue with Hoechst33342.

### Fluorescence microscopy

The cells were observed using an inverted Nikon Eclipse Ti2-E microscope (Nikon Instruments, Tokio, Japan) coupled with an Andor Dragonfly spinning disk unit and a high magnification objective, NIKON 60x NA 1.4, on a high-resolution camera (Zyla 4.2, 2.0 Andor, Oxford Instruments Company, Belfast, UK). Fusion software version 2.4.0.14 (Andor Oxford Instruments Company, Belfast, UK) was used for acquisition as previously reported^6^. Image processing and analysis were performed with Image J/Fifi 1.54 open-source software. For quantitative assessment of sEV uptake, red fluorescence per cell was measured in a minimum of 200 cells per condition using ImageJ/Fiji 1.54.

### Measuring L-lactate concentration on sEV

Lactate association with sEVs was measured in purified vesicle preparations after incubation with lactate using an L-Lactate Assay Kit (Sigma, MAK570) according to the manufacturer’s instructions. All measurements were performed in triplicate.

### Statistical analysis

Statistical analysis was performed with GraphPad Prism and RStudio software. Data are presented as mean ± standard error of the mean (s.e.m.) Data were analysed using a two-tailed Student’s t-test for comparison of two groups, and one-way analysis of variance (ANOVA) analysis followed by Tukey’s post-test when comparing three groups. Statistical significance was considered when *p* < 0.05.

## Results

### Effect of lactate on sEV activity in vitro

**Fig. 1:**
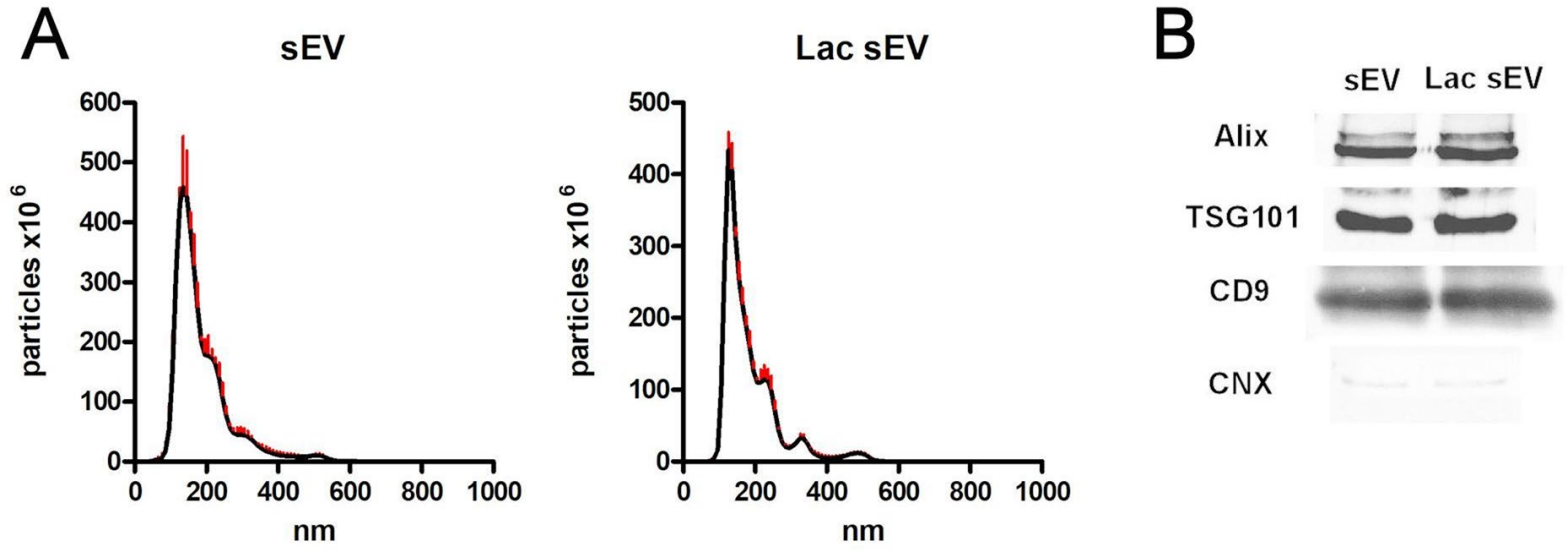
(**A**) Nanoparticle tracking analysis of extracellular vesicles (sEVs) obtained from BxPC-3 cells. Untreated vesicles (sEV) are shown on the left, and vesicles treated with 28 mM sodium lactate (Lac sEV) are shown on the right. (**B**) Western blot showing the presence of TSG101, Alix and CD9 as well the absence of calnexin (CNX).

To assess whether the lactate concentration present in LR influences sEV activity, we first evaluated the effects of vesicles derived from BxPC-3 pancreatic cells (Fig. 1) incubated with 28 mM sodium lactate, corresponding to the lactate concentration in LR. Vesicle characterization by nanoparticle tracking analysis revealed a size distribution consistent with small extracellular vesicles (sEVs), and the presence of canonical exosomal markers detected by Western blotting further confirmed their classification as sEVs. Treatment with lactate did not alter the size distribution of sEVs compared with control vesicles, indicating that the observed effects are not due to gross changes in vesicle characteristics.

### Effect of lactate on sEVs

**Fig. 2:**
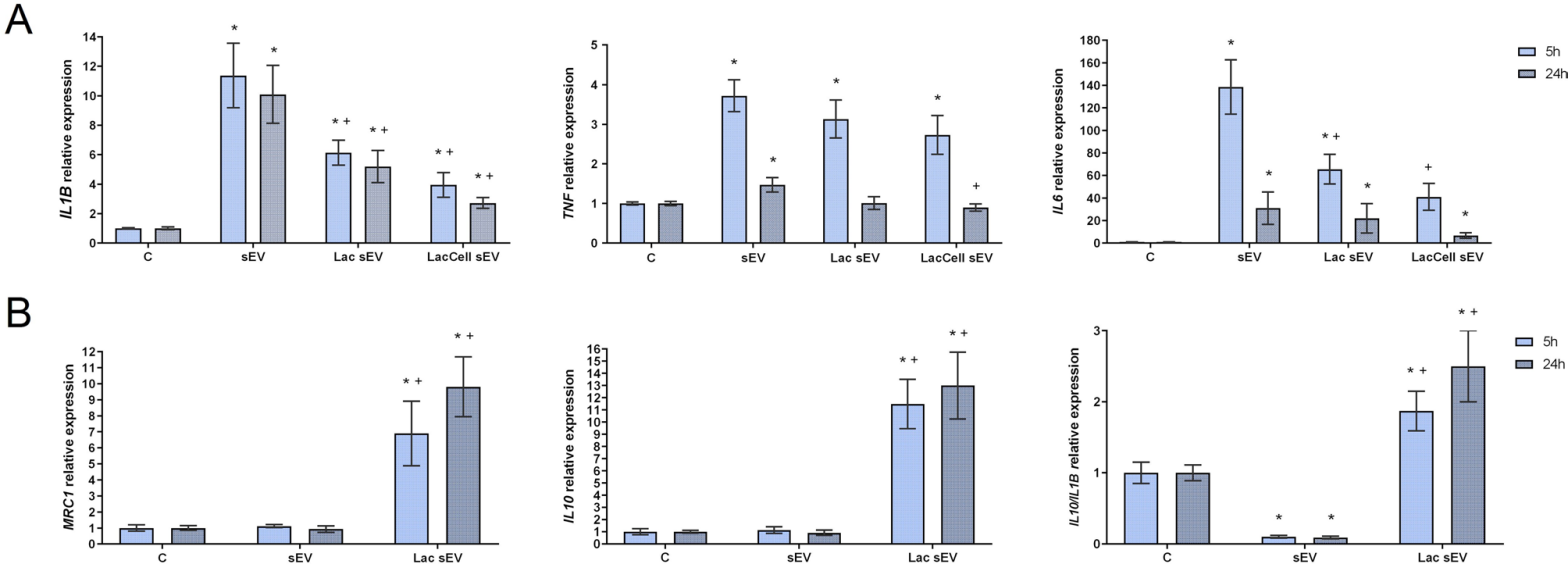
(**A)** Induction of IL1B, TNF, and IL6 RNA expression in THP-1 macrophages following exposure for 5–24 h to vesicles obtained from lactate-treated cells (LacCells sEVs) or to vesicles directly exposed to lactate (Lac sEVs). (**B**) RNA Expression of the M2 markers MRC1 and IL10, and the IL10/IL1 ratio, in THP-1 macrophages exposed to lactate-treated sEVs. *=*p* < 0.05 vs. C; +=*p* < 0.05 vs. sEV.

Macrophages differentiated from THP-1 cells and exposed to BxPC-3–derived vesicles for 5–24 h exhibited a moderate inflammatory response, characterized by increased expression of the pro-inflammatory cytokines IL1B, TNF, and IL6. As vesicles were obtained from BxPC-3 cells cultured in serum-free medium to avoid contamination by serum-derived extracellular vesicles, this moderate inflammatory activity is likely attributable to cellular stress associated with serum deprivation. Supplementation of the BxPC-3 culture medium with lactate during vesicle production significantly attenuated the induction of IL1B and IL6 expression in macrophages exposed to these vesicles (Fig. 2A).

Because these vesicles were generated in the presence of lactate in the BxPC-3 culture medium, a direct effect of lactate on the vesicles themselves, rather than on the producer cells, could not be excluded. To address this possibility, vesicles purified from BxPC-3 cells cultured in lactate-free medium were incubated with 28 mM sodium lactate for 1 h prior to macrophage exposure. Lactate-treated vesicles elicited a similarly reduced inflammatory response compared with vesicles generated under lactate-supplemented culture conditions (Fig. 2A).

Given the comparable effects observed under both conditions, subsequent experiments were performed using direct lactate treatment of purified sEV.

### Effect on macrophage polarization

To determine whether this modulation reflected solely a reduction in macrophage activation or involved changes in macrophage phenotype, we examined markers associated with macrophage polarization. Expression of the M2-associated markers IL10 and MRC1 was increased following exposure to lactate-treated sEVs. Consistently, the M2/M1 ratio, calculated as IL10/IL1B, shifted toward an M1 phenotype in response to untreated vesicles but reverted toward an M2 phenotype upon exposure to lactate-conditioned sEVs (Fig. 2C).

### Stereoisomer specificity

**Fig. 3:**
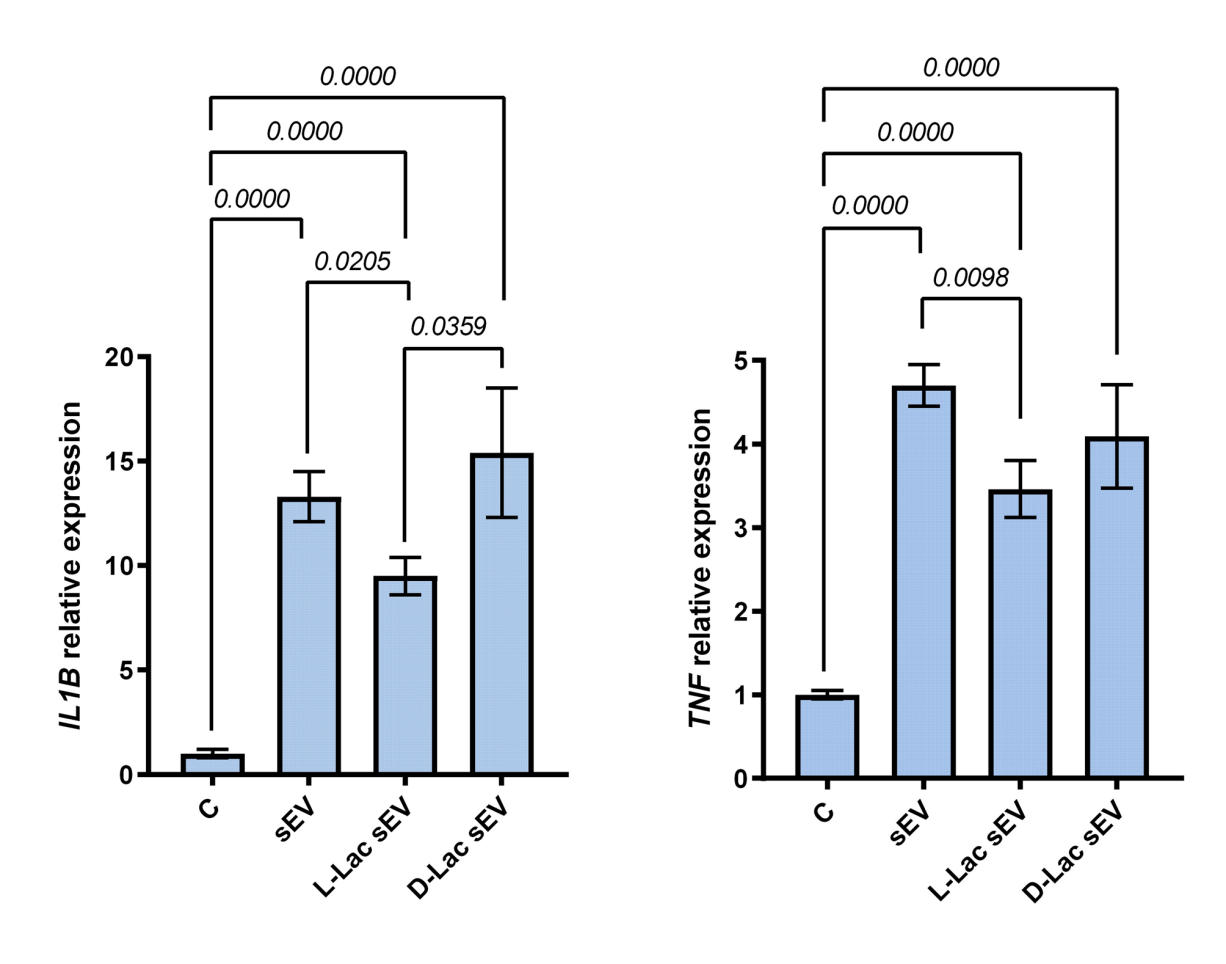
The effects of lactate treatment of sEVs on their ability to induce cytokine expression are dependent on L-lactate, as this isomer was the only one to elicit the effect, whereas D-lactate did not alter the sEV response.

LR contains a mixture of the two stereoisomers of lactate. To determine which isomer was responsible for modulating sEV inflammatory activity, vesicles derived from BxPC-3 cells were treated with 14 mM of either D-lactate or L-lactate. THP-1-derived macrophages were then incubated with these vesicles for 5 h, and IL1B expression was assessed. Treatment with L-lactate, but not D-lactate, significantly attenuated the inflammatory response induced by sEVs (Fig. 3).

### Vesicle uptake by macrophages

**Fig. 4:**
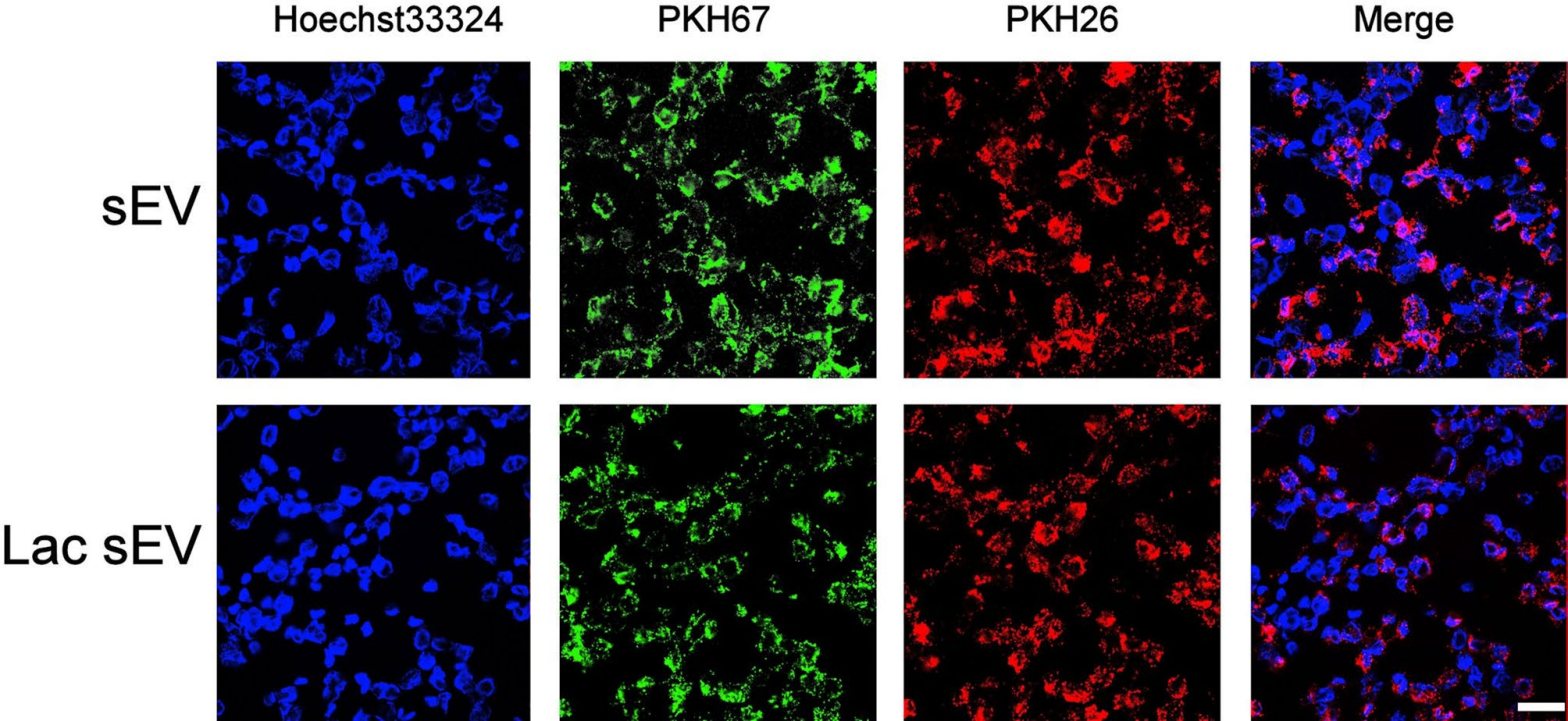
No differences in sEV uptake were observed between control (sEV) and lactate-treated (Lac sEVs) vesicles after 3 h of incubation with THP-1 macrophages. THP-1 cells were stained with PKH67 (green), sEVs were stained with PKH26 (red), and nuclei were stained with Hoechst 33,342 (blue). Scale bar: 20 μm.

One potential mechanism by which lactate could modulate sEV activity is by altering vesicle uptake by target cells. To assess whether lactate affected macrophage uptake, sEVs were labelled with the red fluorescent dye PKH26, and their internalization by THP-1-derived macrophages was evaluated after 3 h of incubation. Imaging analysis revealed no apparent differences in vesicle uptake between lactate-treated and control sEVs and quantification confirmed the lack of changes (sEV 62.3 ± 2.5 vs. Lac sEV 66.2 ± 2.8) (Fig. 4).

### Lactate binding to sEV

To determine whether lactate could be physically associated with sEVs and act merely as a cargo molecule, we measured lactate levels in purified vesicle preparations after incubation with lactate and subsequent washing. Lactate concentrations were below the limit of detection, indicating that lactate-treated sEVs do not carry measurable amounts of lactate.

### Effect of LR on the inflammatory activity of plasma sEVs from patients with acute pancreatitis

**Fig. 5:**
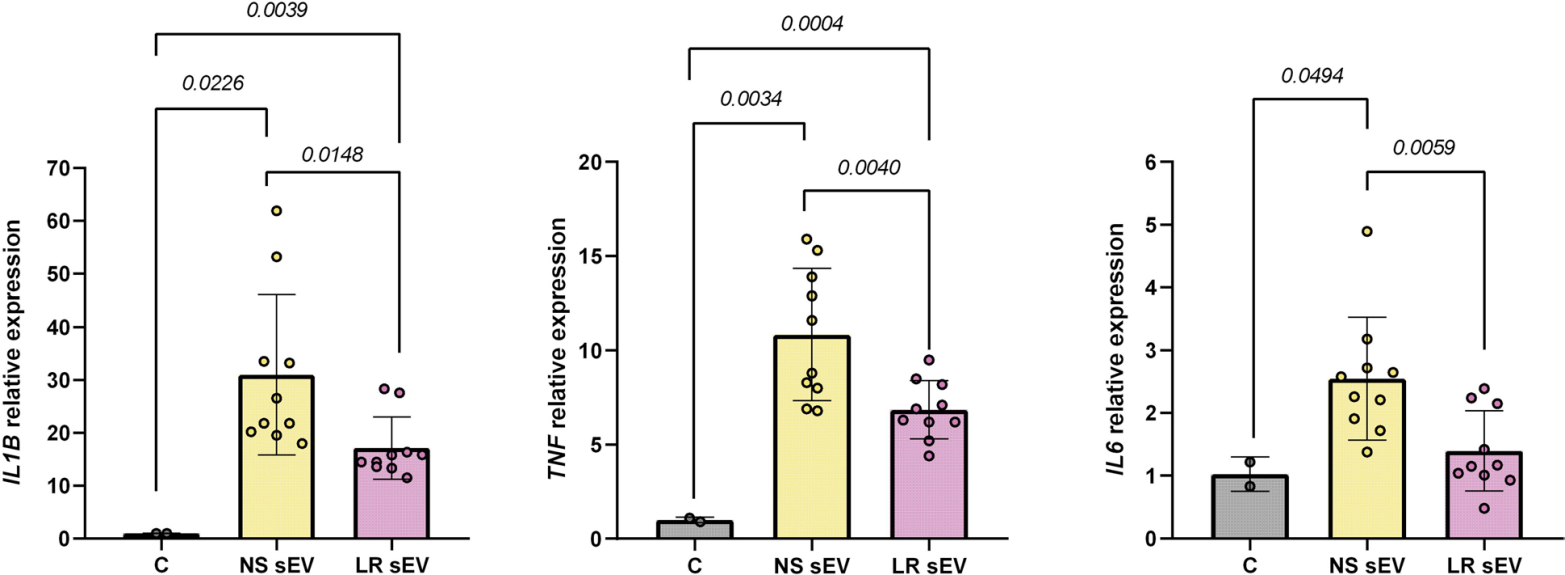
Vesicles obtained from patients with acute pancreatitis who received lactated Ringer’s solution (LR sEV) induced lower expression of IL1B, TNF and IL6 at 5 h in macrophages compared to those from patients who received normal saline (NS sEV).

To assess whether patient treatment with LR influences the inflammatory activity of circulating sEVs, THP-1–derived macrophages were exposed to plasma sEVs isolated from patients with acute pancreatitis treated with LR or NS (*n* = 10 per group). Macrophages not exposed to any sEVs served as the control group (C). sEVs from LR-treated patients displayed a reduced capacity to induce IL1B, TNF, and IL6 expression compared with sEVs from NS-treated patients (Fig. 5).

**Fig. 6:**
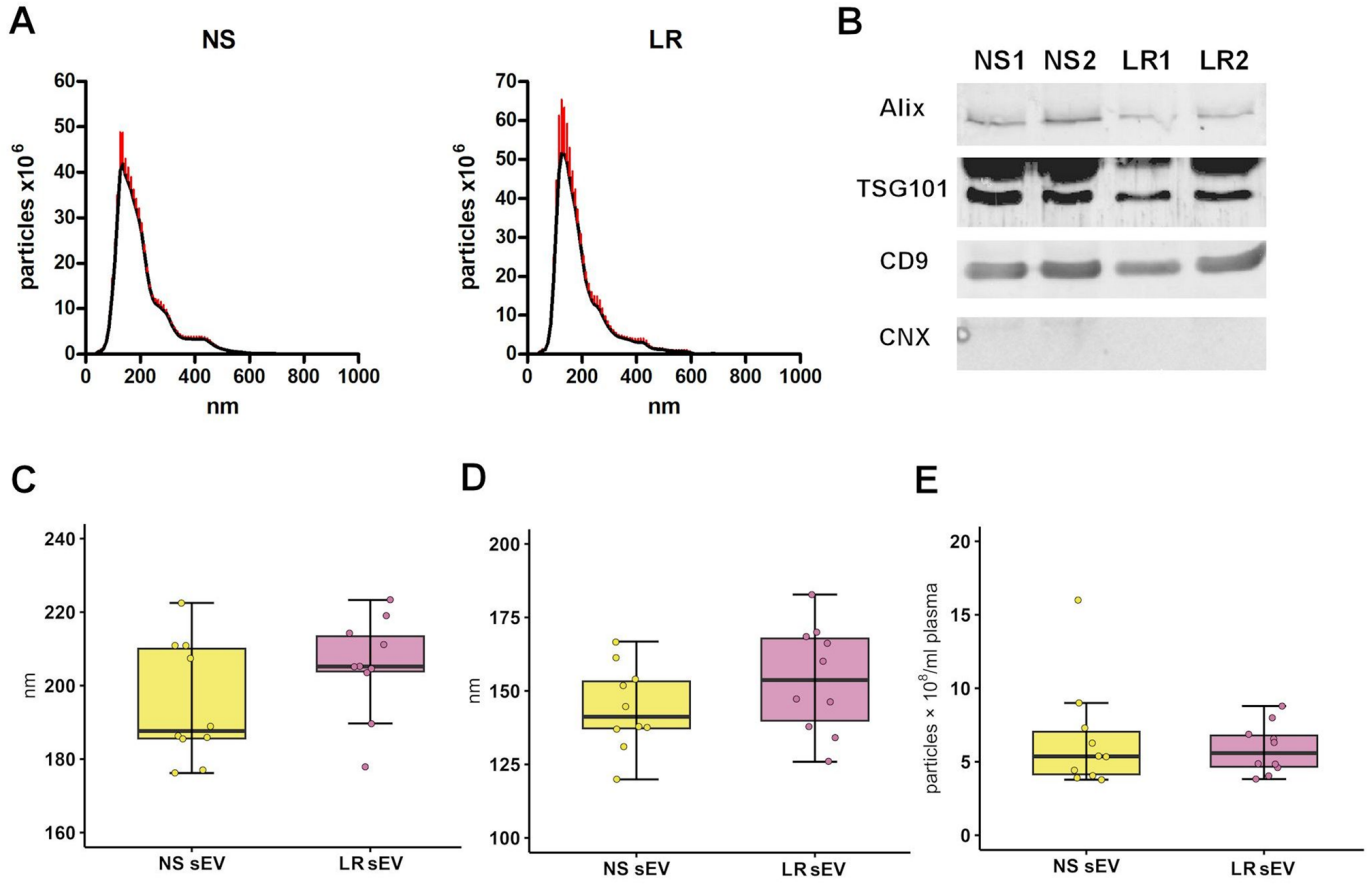
Characterization of plasma sEVs from patients with acute pancreatitis treated with lactated Ringer’s solution (LR sEV) or normal saline (NS sEV). (**A**) Nanoparticle tracking analysis (NTA) of sEVs isolated from 10 NS-treated patients and 10 LR-treated patients. (**B**) Western blot showing the presence of Alix, TSG101, and CD9, and the absence of calnexin (pool of 5 patients per lane). (**C**–**E**) Graphs showing mean sEV size (**C**), mode size (**D**), and concentration (**E**) as determined by NanoSight. No significant differences were observed between the two experimental groups.

Consistent with the in vitro results, sEVs from both patient groups did not differ in size, concentration, or other vesicle characteristics (Fig. 6).

### Lactylation of sEV proteins

**Fig. 7:**
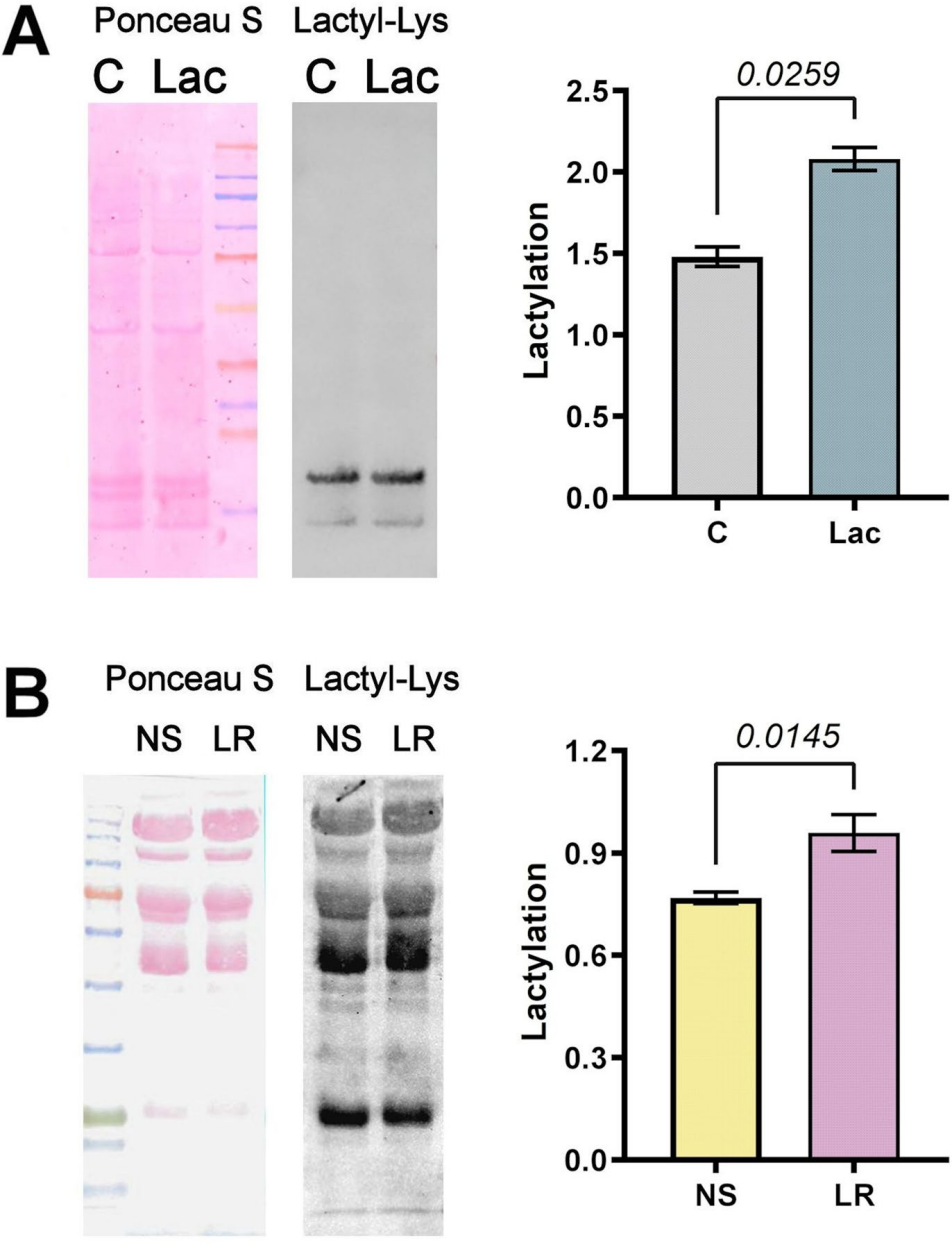
Lactate increases protein lactylation in sEVs in vitro and in patient-derived. (**A**) sEVs isolated from pancreatic BxPC3 cells were incubated in the absence (C) or presence (Lac) of lactate (28 mM). Protein lactylation was analyzed by anti–lactyl-lysine Western blot and signal were normalized to total the corresponding protein staining (Ponceau S). (**B**) sEVs isolated from plasma of patients with acute pancreatitis treated with normal saline (NS) or lactated Ringer’s solution (LR) were analyzed as in (**A**). A modest but consistent increase in protein lactylation was observed in sEVs from LR-treated patients. Data are shown as mean ± SEM.

Since lactylation of constituent proteins is a potential mechanism by which lactate modulates sEV activity, we examined the presence of lactylated proteins in vesicle preparations by Western blot using an anti-lactyl-lysine antibody. Vesicles contained substantial levels of lactylated proteins under basal conditions, and treatment with lactate resulted in a detectable increase. Densitometric analysis of Western blot bands, normalized to total protein staining with Ponceau S, confirmed a modest but consistent increase in the ratio of lactylated protein to total protein in lactate-treated vesicles (Fig. 7A).

We then investigated whether a similar phenomenon occurred in sEVs isolated from patients of acute pancreatitis treated with either NS or LR. As expected, the protein profiles in these clinical samples differed from those of in vitro-derived vesicles. Nevertheless, high levels of protein lactylation were observed in both patient groups. Densitometric analysis revealed a modest but consistent increase in the ratio of lactylated protein to total protein in sEVs from patients who received LR compared with those treated with NS (Fig. 7B).

## Discussion

In this study, we show that lactate at concentrations present in LR modulates the inflammatory activity of small extracellular vesicles (sEVs). In recent years, increasing attention has been focused on the potential advantages of using LR rather than NS during fluid resuscitation in acute pancreatitis^2,3,10,11^. LR administration has been associated with a lower incidence of systemic inflammatory response and reduced C-reactive protein levels compared with patients receiving NS^3,10^.

In a previous study, we demonstrated that the anti-inflammatory effects of LR were linked to the presence of lactate, as removal of this molecule from the solution abolished its inhibitory effect on macrophage activation^3^. However, the mechanisms through which lactate present in LR exerts these effects have remained unclear.

Small extracellular vesicles have been increasingly recognized as contributors to systemic inflammation in a variety of clinical conditions^12^. In the context of acute pancreatitis, the severity of the systemic inflammatory response has been associated with the pro-inflammatory activity of circulating sEVs, likely reflecting their capacity to activate macrophages^6^. On this basis, we investigated whether the anti-inflammatory effects of lactate are mediated through direct modulation of sEV activity.

We initially analyzed the inflammatory activity of sEVs obtained from pancreatic cells and assessed how exposure to 28 mM lactate modulated this activity. This lactate concentration was selected because it corresponds to that present in LR^13^. We observed that sEVs induced an increased expression of inflammatory markers in macrophages (Fig. 2A), and that the presence of lactate attenuated this induction.

The macrophage inflammatory response to these sEVs is likely related to the conditions under which they were obtained, as vesicles were isolated from cells cultured in the absence of FCS. While this approach prevents contamination by serum-derived vesicles, it also induces cellular stress due to nutrient limitation. This stress was reflected in the properties of the released sEVs and we took advantage of this feature to induce a reproducible macrophage activation driven specifically by sEVs.

A limitation of this initial approach was that the effect of lactate could result either from its action on the pancreatic cells producing the sEVs (LacCel-sEV) or from a direct effect on the vesicles themselves. Therefore, we also evaluated the effect of directly incubating purified sEVs (Lac-sEV) with lactate. This treatment resulted in a similar inhibition of inflammatory cytokine induction, indicating that lactate present in LR can directly modify sEVs, reducing their pro-inflammatory capacity (Fig. 3A).

It is important to note that the effect of lactate on sEVs appears to extend beyond a general reduction in macrophage activation. Lactate-treated sEVs (Lac-sEV) not only reduced the expression of pro-inflammatory cytokines such as IL1B and TNF, but also increased markers commonly associated with anti-inflammatory macrophages, including IL10 and MRC1^14^. Although it is accepted that the distinction between M1 and M2 phenotypes is overly simplistic^15^, these observations are consistent with a possible shift toward a more anti-inflammatory activation state rather than a mere suppression of macrophage activity (Fig. 2B).

Another factor we investigated was the lactate stereoisomer responsible for this effect. In LR, the 28 mM concentration corresponds to a racemic mixture of the D- and L-isomers^16^. To determine whether both contributed equally or if the effect was specific to one form, sEVs were treated with 14 mM of each isomer separately. This experiment revealed that the reduction in inflammatory activity was associated with the L-isomer (Fig. 3), an enantiospecificity that may suggest the involvement of stereosensitive mechanisms in this process.

Given this observed effect of lactate treatment, we next explored the potential mechanisms underlying the reduced inflammatory activity. One possible explanation could be that lactate treatment affects the uptake of sEVs. However, this does not appear to be the case, as microscopic tracking revealed no appreciable differences in the number of vesicles internalized by these cells (Fig. 4). Moreover, the observation that lactate-treated sEVs induced the expression of M2 markers further suggests that the mechanism involved is not simply a reduction in vesicle uptake.

We also evaluated whether lactate simply binds to the vesicles, potentially acting as an additional delivery system to the interior of target cells. However, when lactate content was measured after incubating the vesicles, levels were below the detection limit of the assay kit. Given that the sensitivity of the kit is in the micromolar range, it is unlikely that direct binding to sEVs is the mechanism involved.

Although lactate treatment clearly resulted in reduced inflammatory activity of sEVs in vitro, it was important to determine whether a similar effect occurs in a clinical setting. To address this, we analyzed the behavior of sEVs purified from the plasma of patients with acute pancreatitis who had received either NS or LR. The situation differs from the in vitro studies, as the lactate concentration in plasma after LR infusion is lower, but the exposure time is prolonged. Additionally, the stimulus differs, since these sEVs are generated in the context of pancreatic inflammatory pathology, a more intense response than the nutrient deprivation stress applied in vitro. This approach therefore allows a more direct evaluation of how the choice of resuscitation fluid (NS or LR) modulates the inflammatory properties of circulating sEVs under clinically relevant conditions.

Consistent with the in vitro data, sEVs obtained from patients treated with LR induced lower macrophage activation than sEVs from patients who received NS (Fig. 5). Notably, no differences were detected in the characteristics of patient-derived sEVs between the two treatment groups; neither vesicle size nor concentration differed (Fig. 6).

A plausible explanation for the effect of lactate on sEVs is the modification of some of their constituent or transported proteins through lysine lactylation. This posttranslational modification is analogous to the better-known acetylation and has been described to be mediated by the same enzymatic machinery, although additional lactyltransferases as well as non-enzymatic mechanisms have also been proposed^17–19^. Consistent with this possibility, we observed that sEVs exhibit substantial basal levels of lactylation of a few proteins, which increased modestly after direct exposure to lactate in vitro (Fig. 7A). The observation that purified sEVs can undergo lactylation upon direct incubation with lactate raises important mechanistic questions. Future studies should address whether sEVs carry associated lactyltransferase activity, such as p300/CBP or related acyltransferases, and whether such enzymatic machinery is localized to the sEV membrane surface, as part of the protein corona, or within the luminal compartment. In this regard, the observed stereospecificity already suggests the involvement of stereoselective enzymatic machinery rather than purely non-enzymatic chemical modification. Distinguishing between these possibilities, and determining the relative contribution of enzymatic versus non-enzymatic lactylation mechanisms, will be essential to fully understand how extracellular lactate modifies sEV protein composition and function.

Importantly, a similar pattern was observed in the clinical setting. In this case, sEVs displayed a lactylation pattern affecting many more proteins. This is not surprising, since, unlike sEVs obtained in vitro, circulating sEVs are highly heterogeneous and can incorporate different plasma-derived proteins that modify their protein corona. Interestingly, sEVs exposed to lactate, i.e., those from patients who had received LR, also showed a slight increase in protein lactylation compared to those from patients treated with NS (Fig. 7B). Although Western blotting indicated increased global protein lactylation in sEVs from Ringer’s lactate-treated patients, this approach does not provide site-resolved identification of the specific proteins or residues involved. Future studies employing targeted and optimized quantitative lactyl-proteomics workflows could build on the global findings reported here by defining which sEV proteins are lactylated, identifying the modified residues, and assessing their potential contribution to the biological effects associated with Ringer’s lactate treatment.

It is worth noting that, although the magnitude of the increase in protein lactylation of sEVs was modest, a consistent trend was observed both in vitro after direct exposure to lactate and in sEVs isolated from patients treated with LR. However, it is important to take into account that the observed association, while consistent and reproducible across our experimental conditions, remains correlative in nature. Definitively establishing causality would require further extensive additional experiments, including proteome-wide identification of lactylated residues as well as functional intervention studies such as treatment with enzymatic agents capable of removing lactyl groups from lysine residues. In addition, it should be noted that sEV composition, and consequently their lactylation profile, is highly dependent on the cellular source, physiological context, and microenvironment. Different sEV populations arising from distinct cell types or pathological conditions may therefore display substantially different lactylation profiles and functional consequences. This inherent heterogeneity warrants caution when extrapolating our findings beyond the specific experimental and clinical settings studied here, and underscores the need for systematic characterization of sEV lactylation across diverse biological contexts before broader generalizations can be made.

Taken together, our findings identify lactate as a modulator of the inflammatory activity of circulating sEVs and suggest that this effect may be partially mediated through enhanced protein lactylation. The convergence between the results obtained in vitro and those derived from patient samples indicates that the choice of resuscitation fluid can influence the immunomodulatory properties of sEVs during acute pancreatitis. These results suggest a mechanistic basis for the clinical benefits associated with LR and highlight sEV lactylation as a potential pathway linking metabolic cues to systemic inflammation. We are aware that the clinical sample is limited and sEVs in plasma are highly variable. Moreover, lactylation of sEVs may not be the only mechanism involved, and additional lactate‑mediated effects cannot be excluded. Nonetheless, the concordance with in vitro data supports the translational relevance of lactylation-mediated modulation of sEV activity, warranting further studies in larger patient cohorts.

## Supplementary Information

Supplementary Material 1

## Data Availability

The datasets used and/or analysed during the current study are available from the corresponding author on reasonable request.

## References

[1] Zhang, S. et al. Lactate: elucidating its indispensable role in human health. *Mol. Cancer*. **25**, 2. 10.1186/s12943-025-02519-z (2026). 41491598 10.1186/s12943-025-02519-z PMC12771723

[2] Mosquera, F. E. C. et al. Fluid Resuscitation with Lactated Ringer vs. Normal Saline in Acute Pancreatitis: A Systematic Review and Meta-Analysis of Clinical Trials. *Dis. Basel Switz.* **13**, 300. 10.3390/diseases13090300 (2025). 10.3390/diseases13090300 PMC12468465 41002736

[3] de-Madaria, E. et al. Fluid resuscitation with lactated Ringer’s solution vs normal saline in acute pancreatitis: A triple-blind, randomized, controlled trial. *United Eur. Gastroenterol. J.* **6**, 63–72. 10.1177/2050640617707864 (2018). 10.1177/2050640617707864 PMC5802674 29435315

[4] Zhang, D. et al. Metabolic regulation of gene expression by histone lactylation. *Nature* **574**, 575–580. 10.1038/s41586-019-1678-1 (2019). 31645732 10.1038/s41586-019-1678-1 PMC6818755

[5] Zhu, Z. et al. Lactylation at the crossroads of immune metabolism and epigenetic regulation: revealing its role in rheumatic immune diseases. *J. Transl Med.* **24**, 25. 10.1186/s12967-025-07498-9 (2025). 41318504 10.1186/s12967-025-07498-9 PMC12772057

[6] Carrascal, M. et al. Inflammatory capacity of exosomes released in the early stages of acute pancreatitis predicts the severity of the disease. *J. Pathol.* **256**, 83–92. 10.1002/path.5811 (2022). 34599510 10.1002/path.5811

[7] Jiménez-Alesanco, A. et al. Acute pancreatitis promotes the generation of two different exosome populations. *Sci. Rep. Sci. Rep.* **9**, 19887. 10.1038/s41598-019-56220-5 (2019). 31882721 10.1038/s41598-019-56220-5 PMC6934470

[8] World Medical Association. World Medical Association Declaration of Helsinki: Ethical Principles for Medical Research Involving Human Participants. *JAMA* **333**, 71–74. 10.1001/jama.2024.21972 (2025). 39425955 10.1001/jama.2024.21972

[9] Banks, P. A. et al. Classification of acute pancreatitis–2012: revision of the Atlanta classification and definitions by international consensus. *Gut* **62**, 102–111. 10.1136/gutjnl-2012-302779 (2013). 23100216 10.1136/gutjnl-2012-302779

[10] Wu, B. U. et al. Lactated Ringer’s solution reduces systemic inflammation compared with saline in patients with acute pancreatitis. *Clin. Gastroenterol. Hepatol.* **9**, 710–717e1. 10.1016/j.cgh.2011.04.026 (2011). 21645639 10.1016/j.cgh.2011.04.026

[11] Zhao, T. et al. Lactated Ringer’s solution versus saline fluid resuscitation for reducing progression to moderate-to-severe acute pancreatitis: a systematic review and meta-analysis. *Int. J. Surg. Lond. Engl.* **111**, 3467–3480. 10.1097/JS9.0000000000002330 (2025). 10.1097/JS9.0000000000002330 PMC12165476 40085761

[12] Zhao, Y. et al. Novel perspectives on extracellular vesicles in autoimmune diseases: immunogenicity, inflammation, and immune surveillance. *J. Clin. Invest.* **136**, e194715. 10.1172/JCI194715 (2026). 41623176 10.1172/JCI194715 PMC12867162

[13] Alsaadi, N. et al. *Shock Augusta Ga.* ; ;**58**:549–555. 10.1097/SHK.0000000000002009 (2022). 10.1097/SHK.0000000000002009 PMC9789188 36399097

[14] Varin, A. & Gordon, S. Alternative activation of macrophages: immune function and cellular biology. *Immunobiology* **214**, 630–641. 10.1016/j.imbio.2008.11.009 (2009). 19264378 10.1016/j.imbio.2008.11.009

[15] Sica, A. & Mantovani, A. Macrophage plasticity and polarization: in vivo veritas. *J. Clin. Invest.* **122**, 787–795. 10.1172/JCI59643 (2012). 22378047 10.1172/JCI59643 PMC3287223

[16] Kuze, S. et al. Effects of sodium L-lactate and sodium racemic lactate on intraoperative acid-base status. *Anesth. Analg*. **75**, 702–707. 10.1213/00000539-199211000-00008 (1992). 1416121 10.1213/00000539-199211000-00008

[17] Shi, P., Ma, Y. & Zhang, S. Non-histone lactylation: unveiling its functional significance. *Front. Cell. Dev. Biol.* **13**, 1535611. 10.3389/fcell.2025.1535611 (2025). 39925738 10.3389/fcell.2025.1535611 PMC11802821

[18] Tan, Q., Liu, M. & Tao, X. Targeting Lactylation: From Metabolic Reprogramming to Precision Therapeutics in Liver Diseases. *Biomolecules* **15**, 1178. 10.3390/biom15081178 (2025). 40867622 10.3390/biom15081178 PMC12385171

[19] Gaffney, D. O. et al. Non-enzymatic Lysine Lactoylation of Glycolytic Enzymes. *Cell. Chem. Biol.* **27**, 206–213e6. 10.1016/j.chembiol.2019.11.005 (2020). 31767537 10.1016/j.chembiol.2019.11.005 PMC7395678

