# Supplementary material for: Lactate Ringer’s anti-inflammatory effects in acute pancreatitis are potentially mediated by protein lactylation of extracellular vesicles
Source: Sci Rep. 2026 Jun 6;16:25919. doi: 10.1038/s41598-026-56277-z (PMC13487186; doi:10.1038/s41598-026-56277-z)

Figure 1

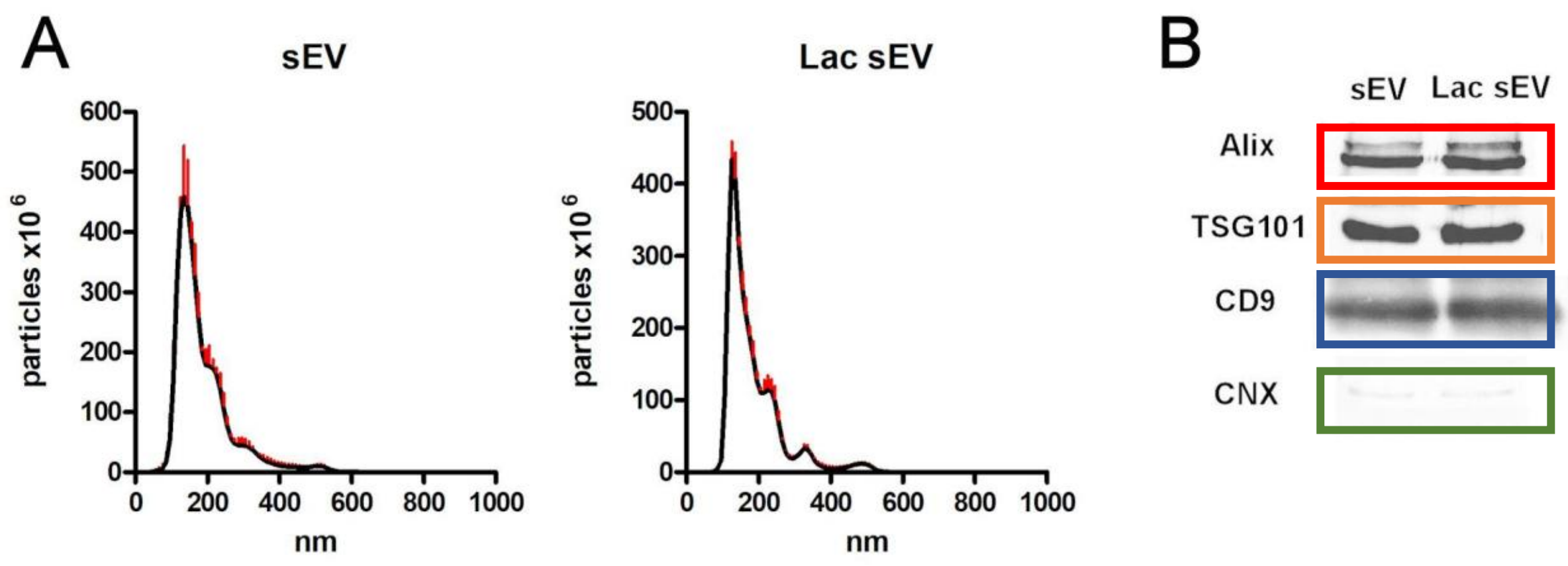

Original plots

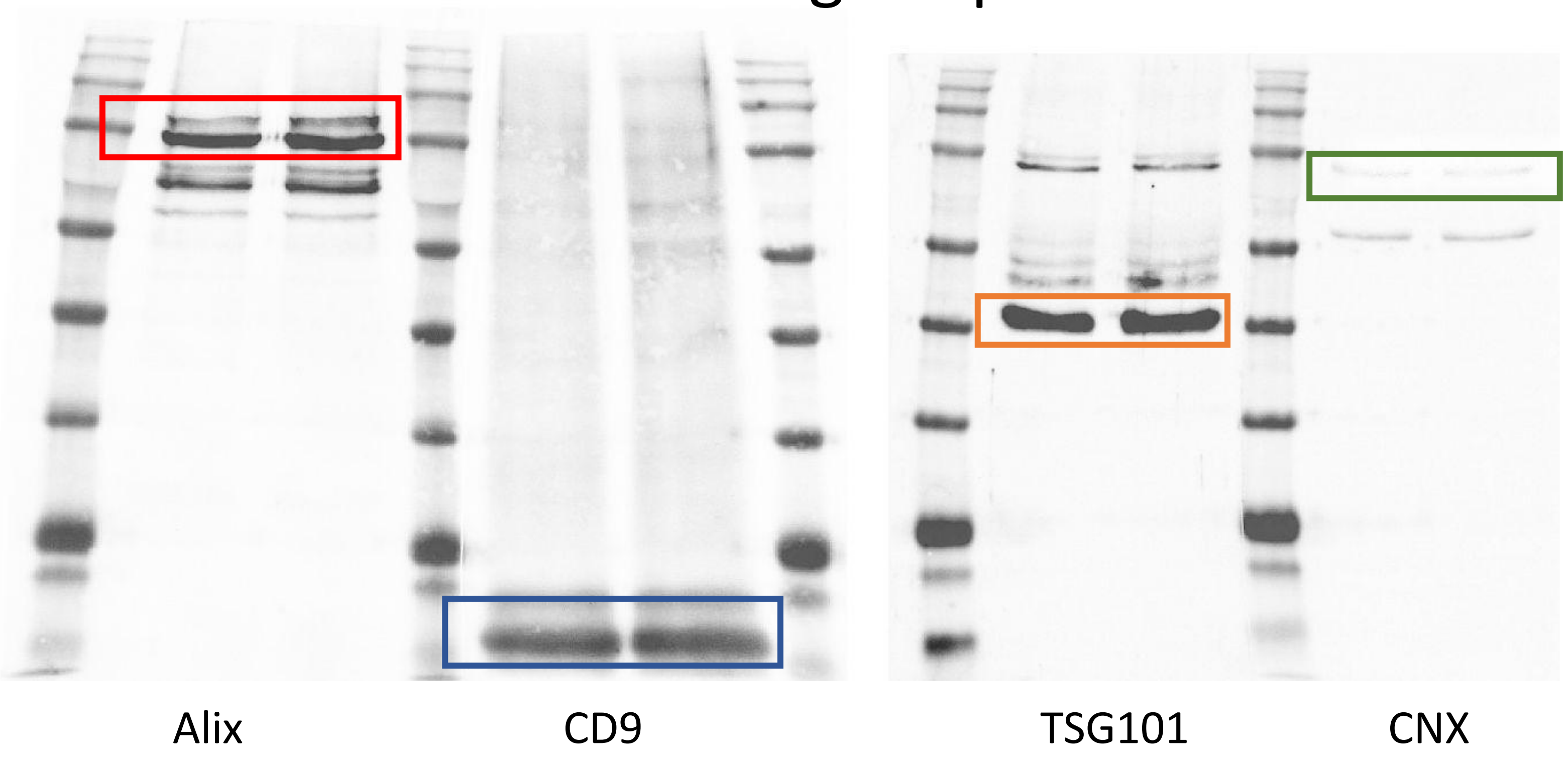

Figure 5

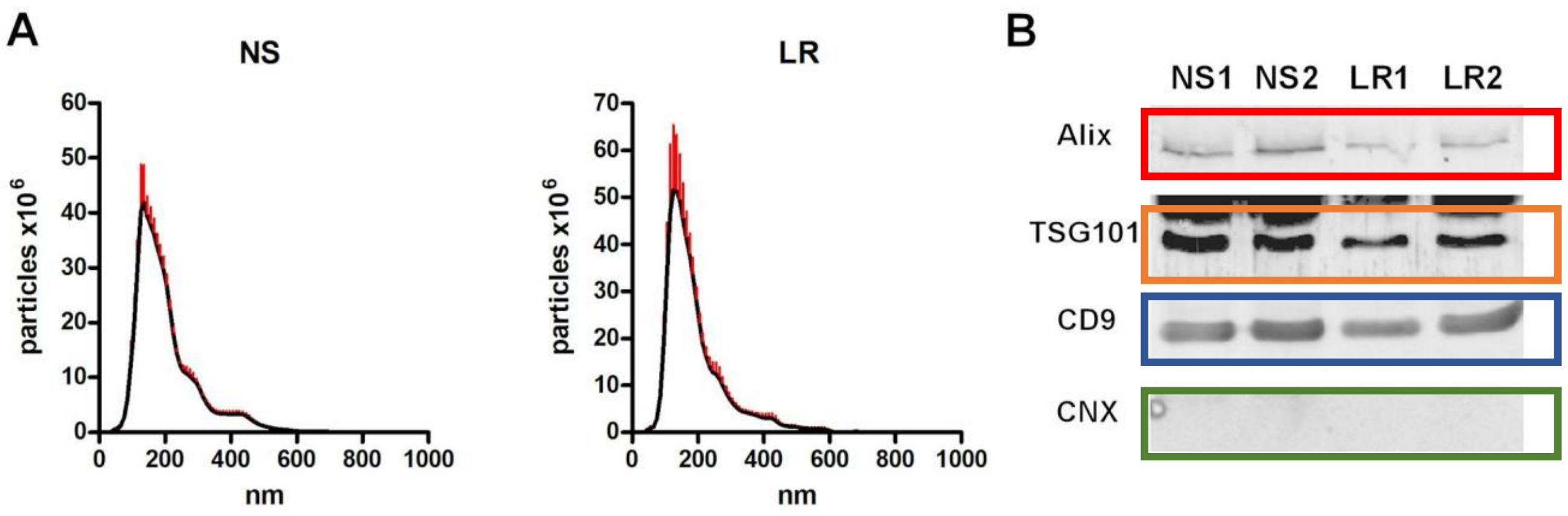

Original plots

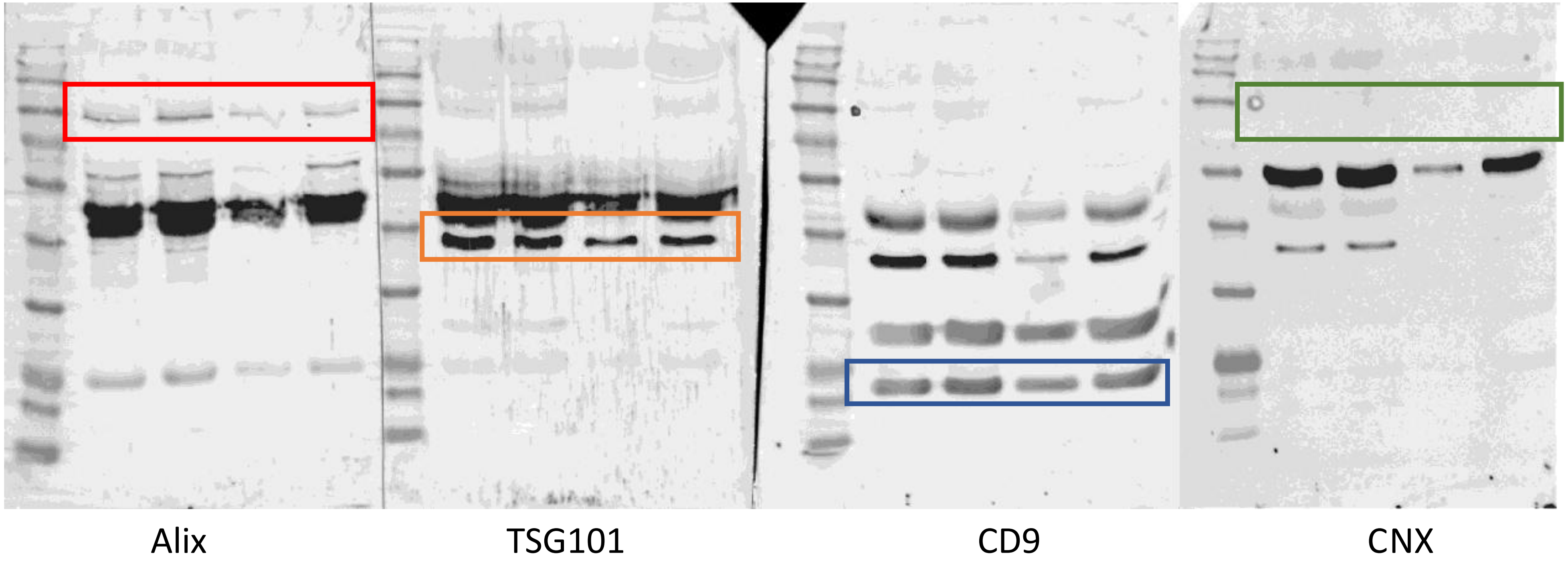

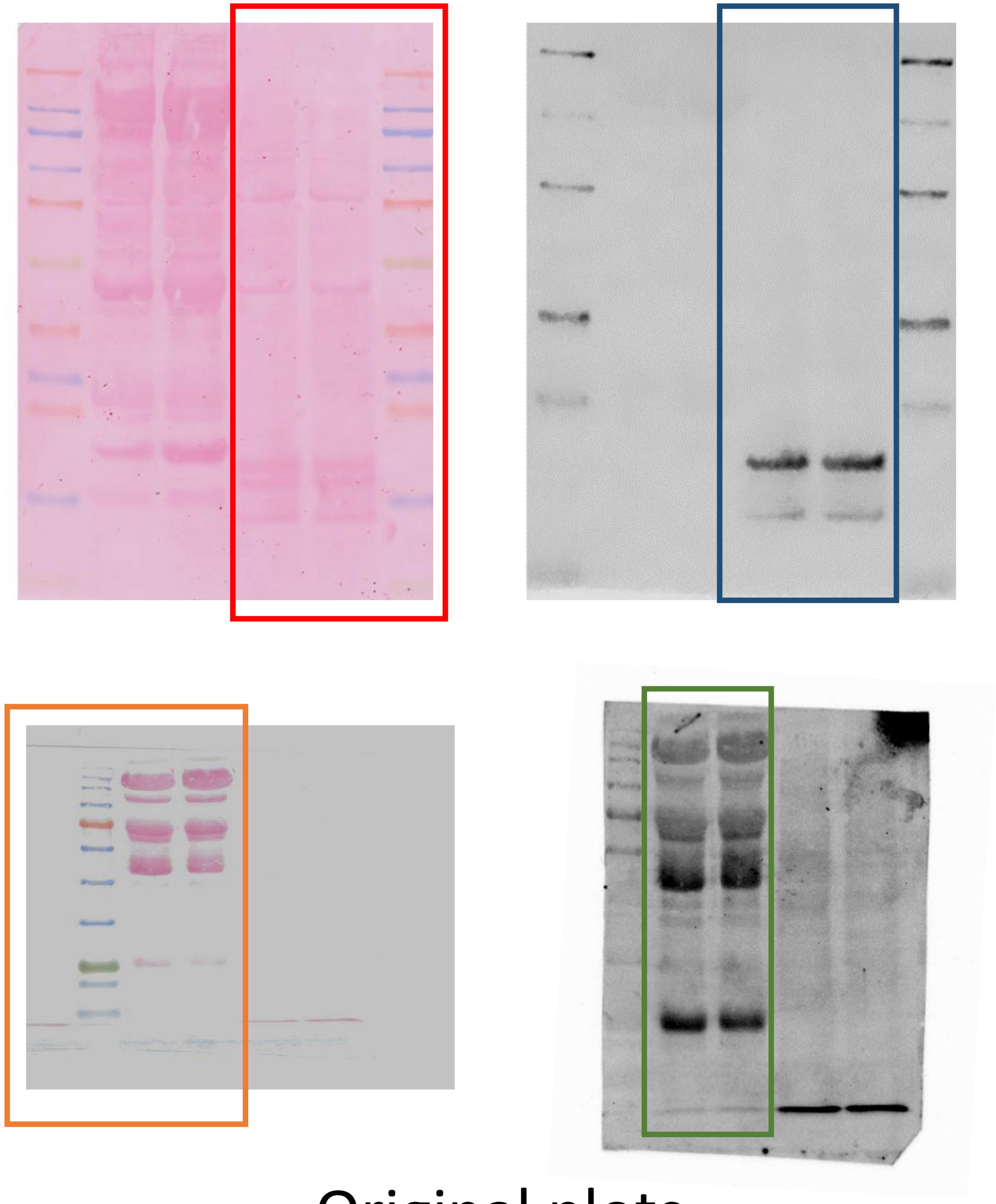

Original plots

Figure 6

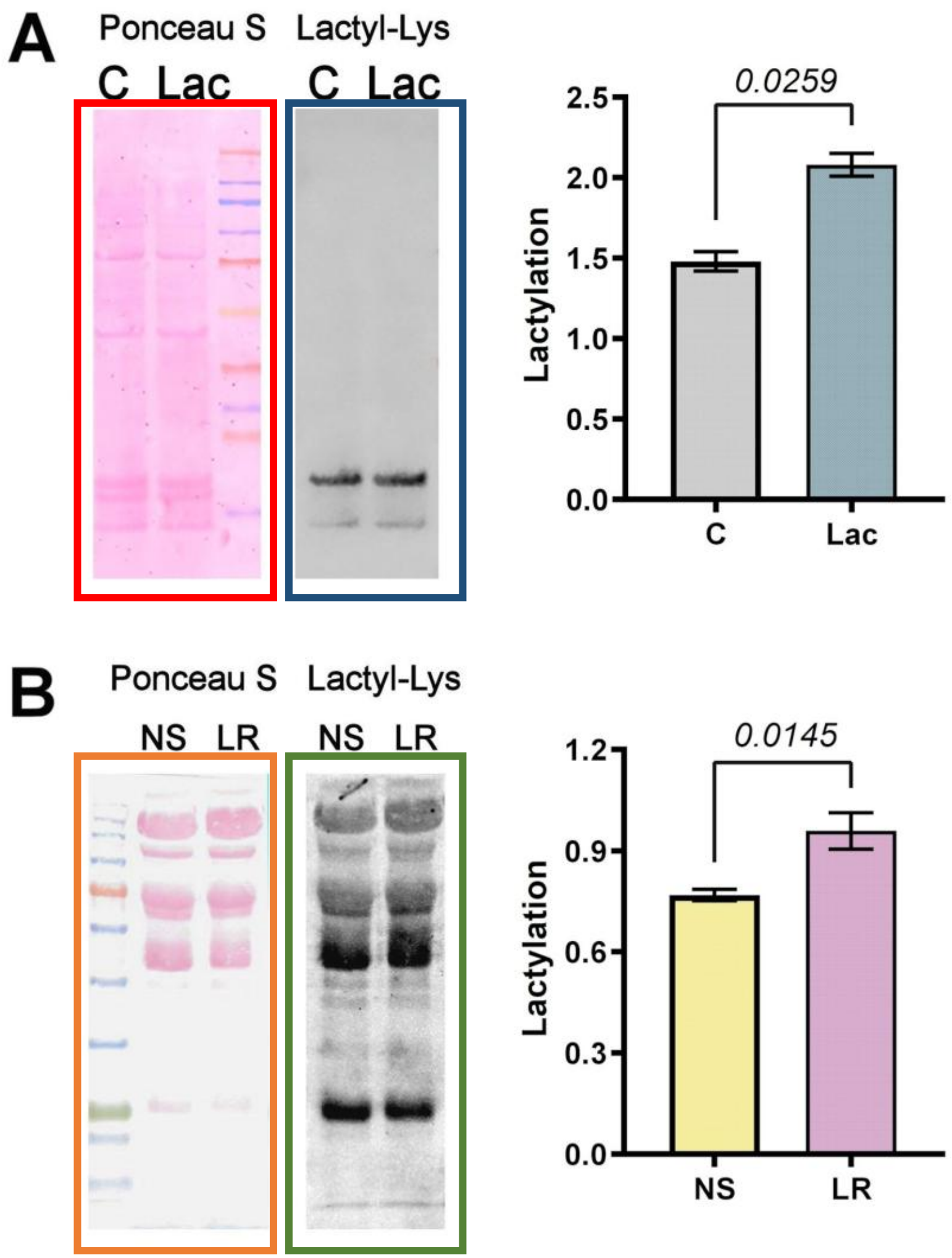

Supplement: Supplementary file 1 [file 41598_2026_56277_MOESM1_ESM.pdf]
